# Complete Genome Sequence of a Renamed Isolate, Trichoplusia ni Ascovirus 6b, from the United States

**DOI:** 10.1128/genomeA.00148-18

**Published:** 2018-03-08

**Authors:** Yuan-Yuan Liu, Wen-Fei Xian, Jin Xue, Yong-Lu Wei, Xiao-Wen Cheng, Xing Wang

**Affiliations:** a Hunan Provincial Key Laboratory for Biology and Control of Plant Diseases and Insect Pests, Hunan Agricultural University, Changsha, Hunan, China; b Institute of Virology, Hunan Agricultural University, Changsha, Hunan, China; c Environmental Horticulture Research Institute, Guangdong Academy of Agricultural Sciences, Guangzhou, Guangdong, China; d Department of Microbiology, Miami University, Oxford, Ohio, USA

## Abstract

The complete genome of Trichoplusia ni ascovirus 6b (TnAV-6b) was sequenced for the first time. The TnAV-6b isolate, which has its closest phylogenetic relationship with the TnAV-6a isolate, has a circular genome of 185,664 bp, with a G+C content of 46.0% and 178 predicted open reading frames.

## GENOME ANNOUNCEMENT

The species Trichoplusia ni ascovirus 6a belongs to the genus Ascovirus, members of which can cause a chronic but ultimately fatal disease in a range of species of Noctuidae, Crambidae, and Plutellidae (1–5). Among 18 isolates of ascoviruses, the genomes of 7 isolates have been sequenced and reported (6, 7). All of these genomes have double-stranded circular DNA of 100 to 200 kb (7–9). In the species Trichoplusia ni ascovirus 6a, only one isolate, TnAV-6a (previously named TnAV-2c), has been sequenced and reported to date (10). Here, we report the complete genome sequence of a new ascovirus member, TnAV-6b (previously named TnAV-2d) (4, 8).

TnAV-6b genomic DNA (4) was sheared for shotgun sequencing to fragment sizes of 400 to 600  bp by ultrasonication and sequenced by the Solexa genome analyzer at the Beijing Genome Institution (BGI; Shenzhen, China). A total of 6,045,713 clean paired-end reads were obtained. Genome assembly was performed using the Velvet assembly software package (11). The assembled contig, representing the entire TnAV-6b genome sequence, was confirmed by six restriction enzyme digestion profiles (BamHI, EcoRI, HindIII, PstI, XbaI, and XhoI) that produced the predicted fragments (4, 8, 12). Open reading frames (ORFs) longer than 150 bp with minimal overlap were considered to be putative genes. Homologies among ascovirus genomes were investigated by using the NCBI Basic Local Alignment Search Tool (BLAST) blastp search (13).

The TnAV-6b isolate has a circular genome of 185,664 bp, with a G+C content of 46.0%, and it encodes 178 predicted ORFs, of which 91 are in the forward orientation and 87 in the reverse orientation. The coding region (168,585 bp) accounts for 90.80% of the total sequences. Fifty-five of the ORFs (30.90%) were annotated with gene functionality predictions. Among the identified ORFs, 163 are related to genes reported for other ascoviruses, including Spodoptera frugiperda ascovirus 1a (SfAV-1a) (42.70%), Heliothis virescens ascovirus 3e (HvAV-3e) (53.37%), HvAV-3f (55.62%), HvAV-3g (55.06%), Diadromus pulchellus ascovirus 4a (DpAV-4a) (25.28%), and TnAV-6a (88.20%). Twenty-seven genes encoding enzymes involved in gene transcription, DNA replication, and nucleotide metabolism were found in the TnAV-6b genome. In addition, 7 baculovirus repeat ORFs (*bro*) and 15 TnAV-6b unique ORFs were found in the genome. TnAV-6b has its closest phylogenetic relationship with TnAV-6a.

Ascoviruses encode apoptosis-related genes to regulate host cell infection (14–16). HvAV-6b encoded 4 inhibitor-of-apoptosis-protein (IAP)-like proteins (ORF3, ORF9, ORF36, and ORF61). ORF3 was 40, 40, 39, and 97% identical, respectively, to the homologous ORFs of HvAV-3e (ORF51), HvAV-3g (ORF55), HvAV-3h (ORF47), and TnAV-6a (ORF3). ORF9 shared 26, 30, 28, 30, 30, and 92% identity, respectively, with the homologous ORFs of SfAV-1a (ORF25), HvAV-3e (ORF28), HvAV-3f (ORF26), HvAV-3g (ORF28), HvAV-3h (ORF25), and TnAV-6a (ORF9), whereas ORF36 was 37, 48, 44, 48, 44, and 99% identical, respectively, to the homologous ORFs of SfAV-1a (ORF74), HvAV-3e (ORF51), HvAV-3f (ORF49), HvAV-3g (ORF44), HvAV-3h (ORF47), and TnAV-6a (ORF32). In addition, ORF61 was 22, 26, 38, 26, 25, and 99% identical, respectively, to the homologous ORFs of SfAV-1a (ORF74), HvAV-3e (ORF51), HvAV-3f (ORF49), HvAV-3g (ORF55), HvAV-3h (ORF47) and TnAV-6a (ORF56), respectively.

### Accession number(s).

The complete genome sequence of TnAV-6b has been deposited in GenBank under the accession no. KY434117.
